# Mapping leaf metal content over industrial brownfields using airborne hyperspectral imaging and optimized vegetation indices

**DOI:** 10.1038/s41598-020-79439-z

**Published:** 2021-01-07

**Authors:** Guillaume Lassalle, Sophie Fabre, Anthony Credoz, Rémy Hédacq, Dominique Dubucq, Arnaud Elger

**Affiliations:** 1 Office National D’Études Et de Recherches Aérospatiales (ONERA), Toulouse, France; 2 TOTAL S.A., Pôle D’Études Et de Recherches de Lacq, Lacq, France; 3 Laboratoire Écologie Fonctionnelle et Environnement (EcoLab), Université de Toulouse, CNRS, INPT, UPS, Toulouse, France; 4 TOTAL S.A., Centre Scientifique Et Technique Jean-Féger, Pau, France

**Keywords:** Plant sciences, Environmental sciences, Optics and photonics

## Abstract

Monitoring plant metal uptake is essential for assessing the ecological risks of contaminated sites. While traditional techniques used to achieve this are destructive, Visible Near-Infrared (VNIR) reflectance spectroscopy represents a good alternative to monitor pollution remotely. Based on previous work, this study proposes a methodology for mapping the content of several metals in leaves (Cr, Cu, Ni and Zn) under realistic field conditions and from airborne imaging. For this purpose, the reflectance of *Rubus fruticosus* L., a pioneer species of industrial brownfields, was linked to leaf metal contents using optimized normalized vegetation indices. High correlations were found between the vegetation indices exploiting pigment-related wavelengths and leaf metal contents (*r* ≤ − 0.76 for Cr, Cu and Ni, and *r* ≥ 0.87 for Zn). This allowed predicting the metal contents with good accuracy in the field and on the image, especially Cu and Zn (*r* ≥ 0.84 and RPD ≥ 2.06). The same indices were applied over the entire study site to map the metal contents at very high spatial resolution. This study demonstrates the potential of remote sensing for assessing metal uptake by plants, opening perspectives of application in risk assessment and phytoextraction monitoring in the context of trace metal pollution.

## Introduction

The assessment of ecological risks deriving from soil pollution has become an essential step in the post-cessation management of industrial activities (mining, oil and gas, etc.). Abandoned industrial lands with high persistent pollution—often termed “brownfields”^1^—are of serious environmental and human health concerns^2–7^. The presence of organic and inorganic pollutants in brownfield soils causes harmful effects on organisms, leading to ecosystem degradation, and imposes selective growing conditions to vegetation, limiting site re-colonization and reshaping existing plant communities^8–11^. Hence, an important effort is required to assess the ecological risks deriving from these sites and to propose relevant methods of remediation during the post-cessation management process^2,12,13^.

Along with organic contaminants, heavy metals (HM, also termed *Trace Metal Elements*) are the main compounds found in vegetated brownfield soils^13–15^. They transfer easily to plant tissues (roots, shoots and leaves), causing oxidative stress and affecting plant photosynthetic capacity, water status, and reproduction^5,16–18^. HM are a serious limitation to the establishment of wild plant species on industrial brownfields^9^. Conversely, several species cope with HM pollution thanks to tolerance, avoidance, and detoxification mechanisms^7,19,20^. Those species capable of accumulating and sequestering HM are usually exploited in phytoextraction trials^2,13^. One common need in assessing HM toxicity and removal by phytoextraction is the estimation of metal uptake by plants. This is traditionally achieved by performing destructive chemical analyses on plant parts. Recently, Visible Near-Infrared (VNIR) spectroscopy proved a reliable, non-destructive tool for monitoring soil pollution in vegetated areas^21–25^. Promising approaches based on tracking alterations in the reflectance of crops and wild plant species have been proposed^15,26–31^. To date, their application remains limited to detecting and quantifying HM concentration solely in soils, which is insufficient in the scenario of brownfield rehabilitation and phytoextraction.

VNIR spectroscopy does not penetrate the ground and is therefore unsuited for estimating HM accumulation in plant roots. Conversely, according to a few recent studies^32–35^, their translocation in plant leaves might be assessed with spectroscopy. The direct detection of HM from leaf reflectance remains very challenging, due to their weak absorption features in the VNIR domain^36^. However, because of their effects on plant biophysical and biochemical properties, HM may be detected indirectly using, for example, pigment-related wavelengths^28,30,32^. Pigments are affected by many other environmental factors, which makes their influence difficult to distinguish from that of HM. In this respect, few attempts succeeded in estimating leaf HM using proximal reflectance measurements in the field^32,33,37,38^. To our knowledge, no study has upscaled this approach to airborne or spaceborne optical imaging, in order to map HM uptake in leaves. This might be helpful for assessing the ecological risk deriving from industrial brownfields and for a non-destructive monitoring of phytoextraction, at large scale. To this end, airborne hyperspectral imaging, which provides reflectance data over narrow and contiguous wavelengths, already proved suitable for developing custom vegetation indices (i.e. reflectance ratios) related to specific plant traits for various applications.

This study aims at estimating the content of several HM in plant leaves using hyperspectral reflectance data, from the field level to airborne imaging. The proposed methodology relies on developing optimized vegetation indices for each metal at the leaf level, before assessing their suitability for use at canopy scale and on airborne images at very high spatial resolution. This study was carried out under realistic field conditions, on naturally established vegetation.

## Results and discussion

### Soil and leaf HM contents

The analysis of soil samples revealed severe and heterogeneous HM pollution of the brownfield site. Cr, Cu, Ni and Zn were found at concentrations up to 3100, 130, 200 and 6100 mg kg^−1^, respectively (Table 1), but these values strongly varied within the site (17% ≤ CV ≤ 42%). HM concentrations were largely higher than the local geochemical background values, and greatly exceeded those observed on the control soil, as well as the environmental recommendations of the European Union (EU) and the French National Institute for Industrial Environment and Risks (INERIS) (except for Ni; see the Supplementary Tables S2 and S4). Therefore, these concentrations were explained by the past industrial activity on the site. In comparison, previous studies involving bramble and HM-polluted soils depicted Cu, Ni and Zn concentrations ranging from 9.7 to 2210, from 12.43 to 53.4, and from 4.87 to 2200 mg kg^−1^, respectively, with maximum values reached on past urban and industrial sites^39–42^. This highlights the strong level of pollution of the study site and the associated ecological risks for natural vegetation and wildlife.

**Table 1:** Heavy metal contents observed in the soil and in the leaves of *R. fruticosus* L. on the brownfield site (*CV* coefficient of variation).

	Range	Mean (± SD)	CV (%)
**Soil (mg kg**^**−1**^**)**			
Cr	1600–3100	2022.5 (± 345.29)	17.07
Cu	56–130	72.9 (± 16.14)	22.14
Ni	55–200	92.55 (± 38.7)	41.82
Zn	3100–6100	4320 (± 836.11)	19.35
**Leaves (mg kg**^**−1**^**)**			
Cr	1.14–3.04	2.14 (± 0.51)	23.83
Cu	10.13–27.91	17.44 (± 5.91)	33.89
Ni	0.53–2.64	1.71 (± 0.44)	25.73
Zn	45.21–149.12	86.91 (± 24.27)	27.93

**Figure 1:**
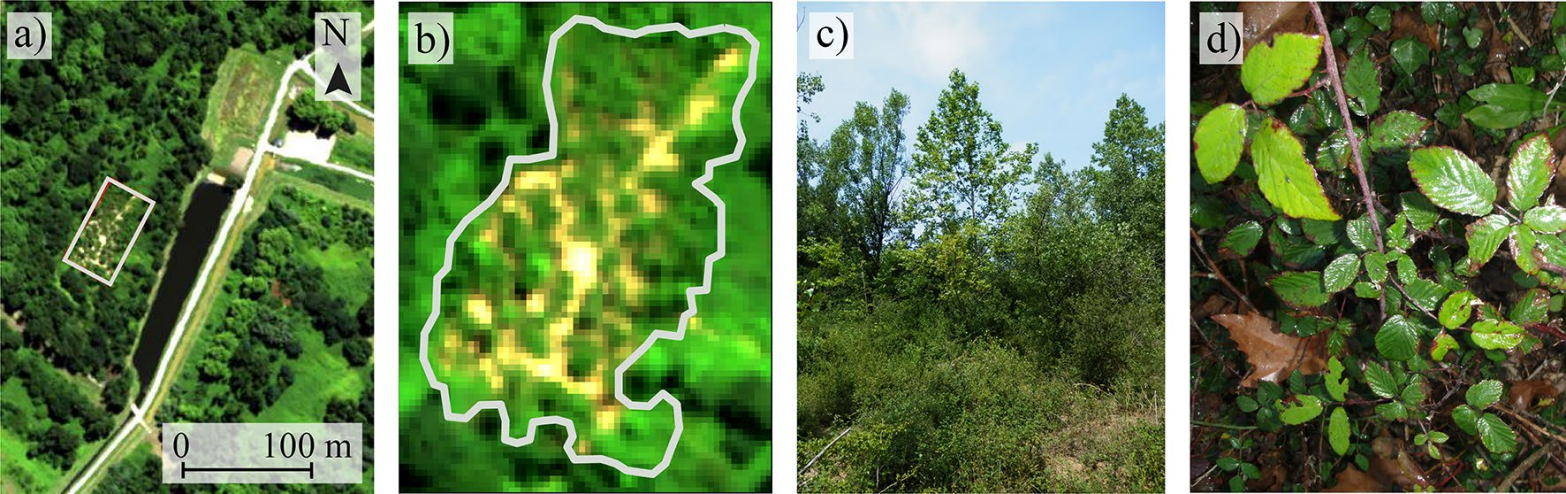
(**a**) Location of the study site on the airborne hyperspectral image acquired with the VNIR-1600 HySpex sensor (R: 647.29 nm, G: 552.77 nm, B: 443.72 nm). This image was generated under Python 3.7.6 ( available at https://www.python.org/). (**b**) Zoom on the study site. The grey polygon shows the delineation of the brownfield obtained from GPS coordinates data. (**c**) The brownfield during the field sampling campaign. The site is mainly colonized by *R. fruticosus* L. (bramble), and bordered by tree vegetation. (**d**) Bramble leaves showing slight symptoms of discoloration and red pigmentation on margins.

Despite the fact that *R. fruticosus* L. typically grows on urban and industrial brownfields, its tolerance to pollution remains limited when exposed to mixtures of contaminants (HM, hydrocarbons, etc.)^30,41,43^. This was expressed visually on the site by a low development and ground cover, and symptoms of stress on leaves (Fig. 1c,d). The four HM contents of bramble leaves were consistent with previous observations made on the same species on other locations^39–42^ (Table 1, see also the Supplementary Table S3). Their variations in leaves within the site ranged from 23.8 to 33.9%, which was favorable to the development of robust prediction models for airborne imaging.

**Figure 2:**
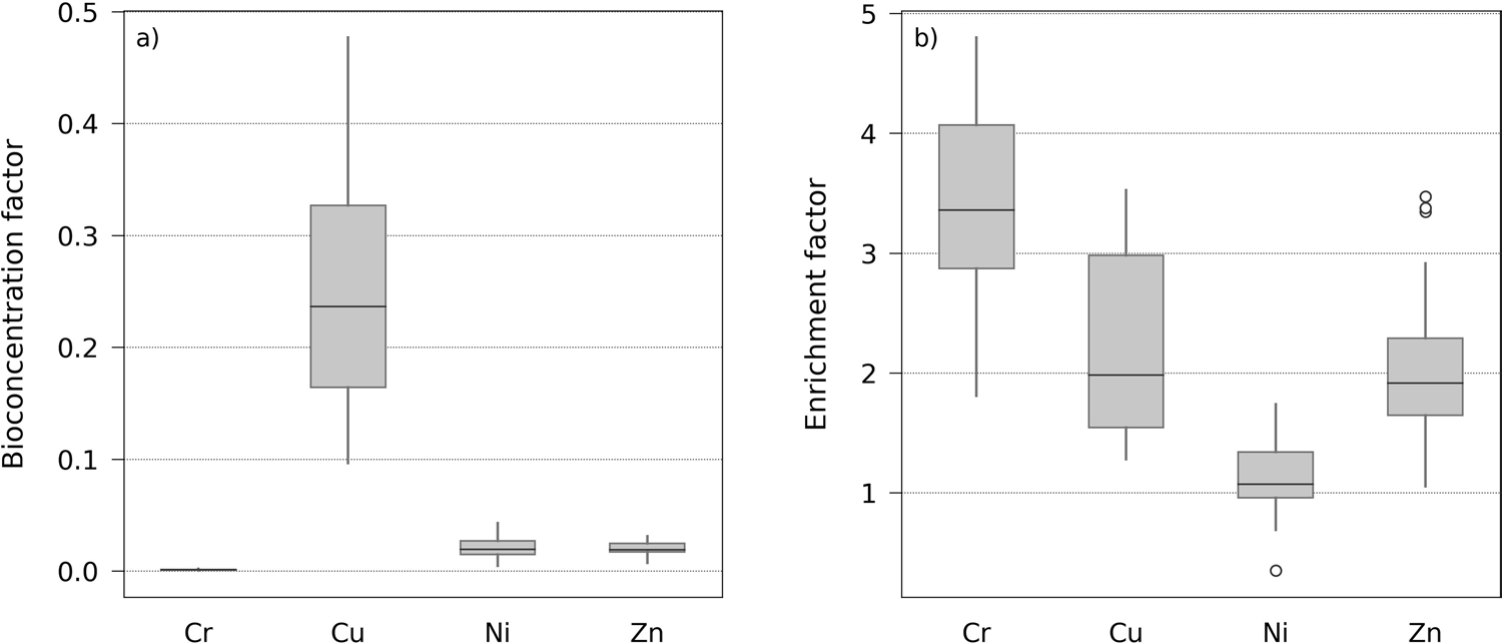
(**a**) Leaf bioconcentration factors (BCFs) and (**b**) enrichment factors (EFs) of *R. fruticosus* L. for the four heavy metals analyzed on the brownfield.

HM uptake and translocation is usually low for bramble^39,40,42^, so only a small fraction of root metal uptake from the soil accumulates in leaves. Therefore, the overall leaf bioconcentration factor (BCF) values were relatively low on the brownfield in comparison to those described for bramble and other plant species in previous studies (Fig. 2a)^2,4,44,45^. Only Cu showed BCF values greater than 0.1 and up to 0.47. This highlighted that bramble is not an hyperaccumulator of HM as defined in recent reviews^2,13^.

Numerous studies have aimed at identifying the mechanisms of HM uptake, translocation and accumulation in plants. These mechanisms have been reviewed recently^7,16^, including those specific to Cr, Cu, Ni, and Zn^18,46–48^. HM uptake involves adsorption on root surface and passive penetration in root tissue via water streams. Then, HM translocate to aerial plant parts through xylem vessels, and accumulate in leaves via symplastic and apoplastic transport^7^. HM accumulation in leaves may also come from atmospheric deposition and uptake through stomata and cuticles^7^, but the underlying mechanisms remain poorly documented and remain specific to a few metals and plant species. Authors point out that HM mobility and uptake by plant roots is influenced by many factors, especially metal speciation, soil properties, and the presence of other contaminants^16,49,50^. For instances, Cr(VI) is much more mobile and toxic to plants than Cr(III)^18,51^. Changes in soil properties—especially pH—affect Cu mobility and uptake by plants^52,53^. Likewise, organic carbon and clay contents—which were very high in our study site—influence Zn and Ni mobility in soils and accumulation in leaves^47,54,55^. In addition, Nie et al*.*^56^ showed that modifications in HM distribution and availability in soils occurs when mixed with petroleum hydrocarbons, such as on our study site. All these factors may account for the low BCF values observed here, explaining the strong tolerance of bramble to high pollution levels.

Although HM are essential to the plant metabolisms—except Cr, they become toxic above a certain concentration that depends mainly on the species and its growing stage, and on the metal type and speciation^5,16–18,30,56^. The enrichment factor (EF) values confirmed that the metal contents of bramble leaves observed on the brownfield were above those normally observed on natural, uncontaminated sites, and thus probably exceed the physiological needs of this species (Fig. 2b). Leaf Cr, Cu and Zn contents were up to 3.5 times higher on the brownfield than on the control site. Only Ni showed almost similar contents between the two sites (0.5 < EF < 1.5). These observations confirmed the suitability of bramble as a model species to develop the methodology of HM estimation by remote sensing.

### Correlation between single-band reflectance and HM

**Figure 3:**
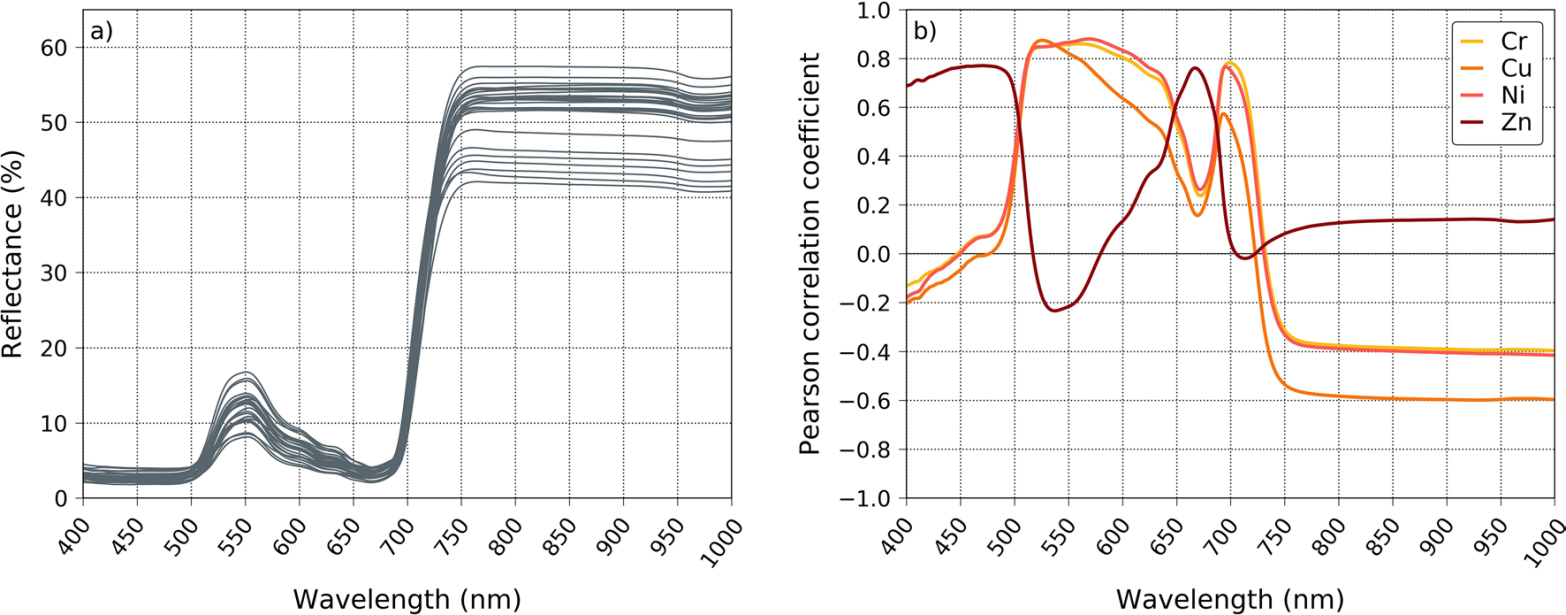
(**a**) Spectral signatures of the leaves sampled on the brownfield (*n* = 26), in the Visible Near-Infrared domain (VNIR). These signatures were used to develop the optimized vegetation indices. (**b**) Pearson correlation coefficients (*r*) obtained between leaf reflectance measured at each VNIR wavelength and the four heavy metal contents analyzed in leaves.

The reflectance of leaves and its single-band correlation with the four HM are presented in Fig. 3. The reflectance of bramble showed the highest variability in the regions of green (~ 550 nm), red-edge (~ 700 nm) and Near-Infrared (NIR) (750–1000 nm) bands. These regions were the most correlated to three out of the four HM contents in leaves—namely Cr, Cu and Ni. More precisely, the green and red-edge bands, which are negatively correlated to chlorophyll and carotenoid contents, were positively correlated to these metals (*r* ≥ 0.87, *p* < 0.001). This suggests the higher the metal uptake, the higher the reflectance and the lower the pigment content in the plant. Such relationship is steadily observed for bramble and other species growing on polluted soils^9,57–59^. In our case, it might be explained by the effects of these three HM on plant metabolisms and biochemistry. Cr, Cu and Ni mostly affect plant pigment contents, involving various mechanisms, such as the replacement of Mg^2+^ in chlorophyll by Cu^2+^ and Ni^2+^ ions, which totally inhibits photosynthesis^16,60^. Cr also alters leaf chlorophyll and carotenoid contents through disorganization of chloroplast ultrastructure. In addition, synergistic, antagonistic and additive effects have been observed under exposure to realistic HM mixtures^30,60^. All these metals also impact plant-water relations negatively at high concentration in soils, affecting leaf turgor and causing tissue destructuring^3,17^. Additional effects on parenchyma structure are also due to the accumulation of these metals in leaves^5,61^. Since leaf anatomy is closely linked to reflectance in the NIR, this probably explains its correlation with HM in this region.

Unlike the other metals, Zn content in leaves was correlated to the blue (400–500 nm) and red (650–670 nm) regions. An increase in reflectance around 650 nm is usually attributed to leaf redding, which comes from anthocyanin synthesis^62,63^. Zn toxicity includes the alteration of photosynthetic pigments through the formation of [Zn]-chlorophyll, which—in contrast to Cu and Ni—still allows photosynthesis. There is also little evidence that Zn induces leaf redding, by enhancing anthocyanin production. This latter effect might prevail over that of other pigments in bramble, explaining the singular pattern of correlation between leaf reflectance and this metal.

### HM estimation using optimized vegetation indices

**Figure 4:**
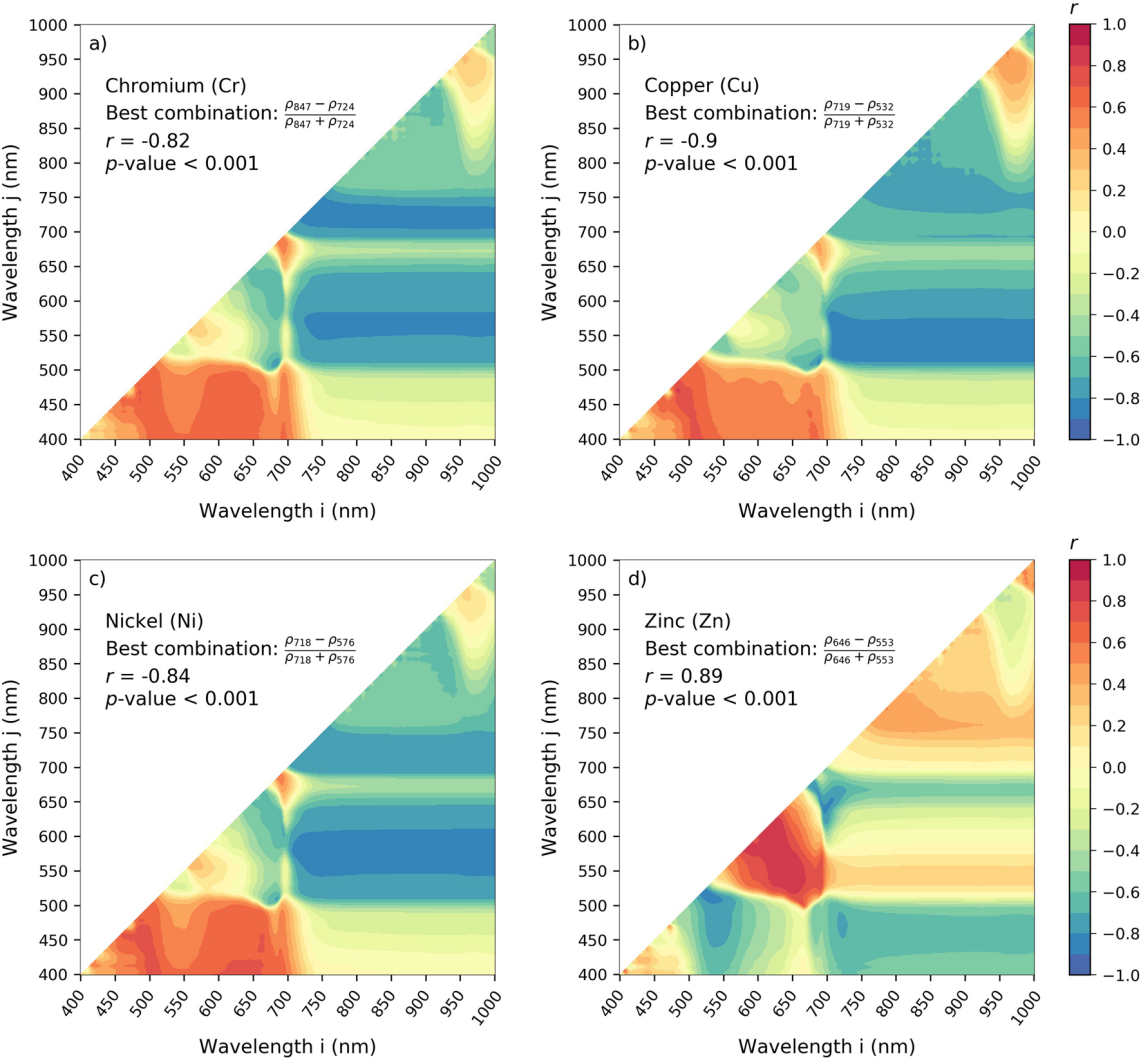
(**a**–**d**) Correlation maps obtained between all possible combination of wavelengths through the normalized vegetation indices and the four heavy metal contents in leaves (*n* = 26). For each combination of wavelength *ρ*_i_ and *ρ*_j_, the normalized index was computed according to Eq. (3), and its correlation with the heavy metal contents was assessed through the Pearson coefficient of correlation (*r*) at the significance level of *p* < 0.05. The best—optimized—index obtained for the four heavy metals was then retained for prediction purposes. The deep blue and red regions show the highest correlations.

The correlations described in “Correlation between single-band reflectance and HM” emphasized the potential of single-band reflectance to estimate leaf HM contents. The next step of the methodology relied on assessing the correlation between all possible two-band combinations in Normalized Difference Vegetation Indices (NDVI-like indices) and each of the four metal contents, still at leaf level. These results are presented in Fig. 4. Generally, the combinations showing high correlations (*r* ≤ − 0.8 or ≥ 0.8) were consistent with the trends observed in “Correlation between single-band reflectance and HM”. The best indices involved bands linked to leaf anatomy (NIR), chlorophyll and carotenoids (green and red-edge) for Cr, Cu and Ni (Fig. 4a–c), and bands linked to the same pigments and also anthocyanins (red) for Zn (Fig. 4d). This was consistent with the study of Zhou et al*.*^32^, who linked Cu and Ni accumulation in leaves to optimized vegetation indices for various species in the field. Likewise, Wang et al*.*^37^, Liu et al*.*^38^, and Zhang et al.^35^ found that the bands related to pigments perform well for estimating other metal contents in plant leaves.

**Figure 5:**
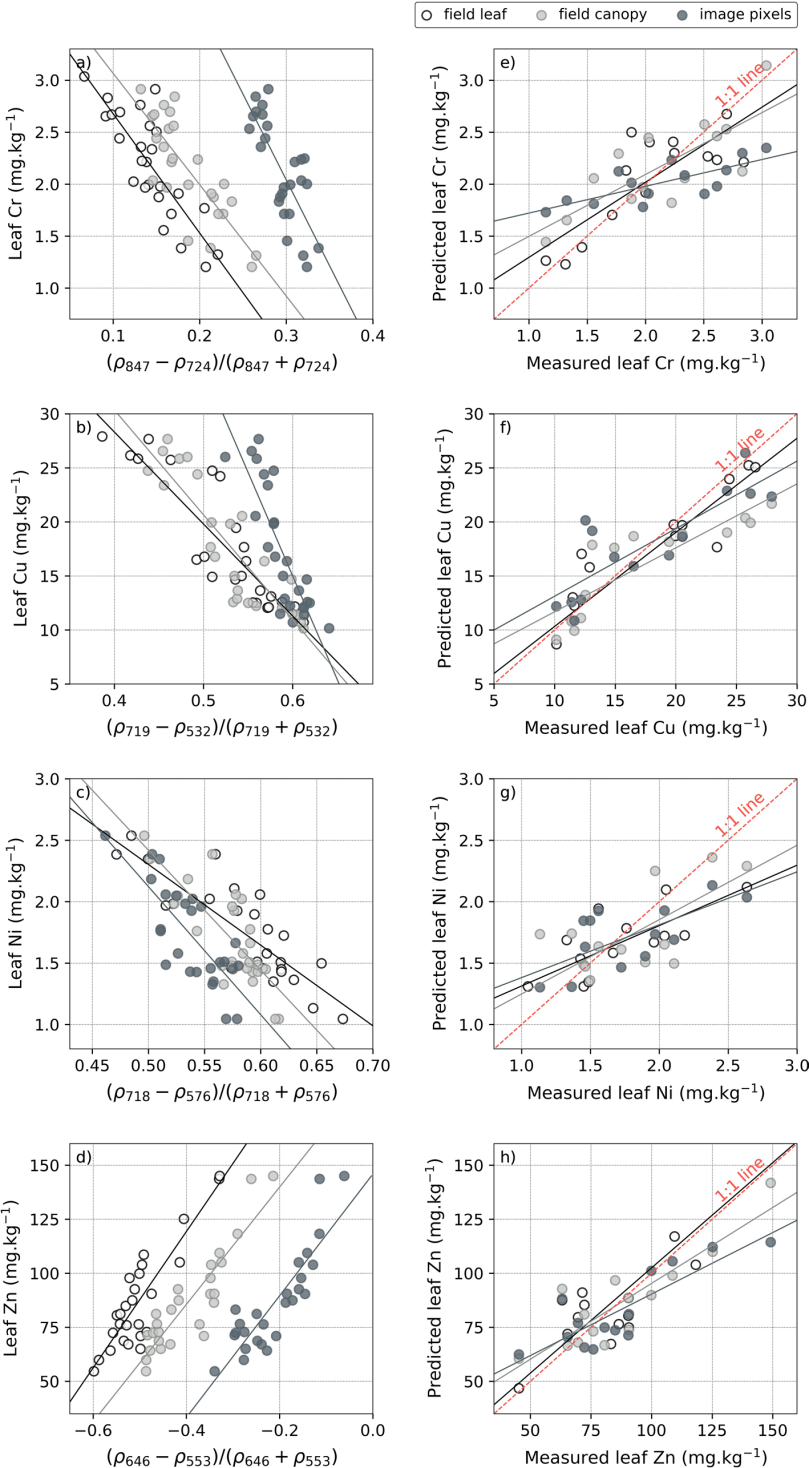
(**a**–**d**) Relationship between the optimized vegetation indices computed at leaf, canopy and image levels, and the four heavy metal contents analyzed in leaves (*n* = 26 at each level). These data were used to calibrate coefficients of linear univariate regression equations, which were thereafter applied to the validation set for predicting heavy metal contents. (**e**–**h**) Comparison between the measured and predicted four heavy metal contents in leaves obtained with the optimized vegetation indices at leaf, canopy and image levels (*n* = 14 at each level). See Table 2 the for the detailed regression equations, correlation coefficients (*r*) and prediction assessment metrics (RMSE, RPD).

Here, the best—optimized—indices were negatively correlated to Cr, Cu and Zn (*r* ≤ − 0.82), and positively to Zn (*r* = 0.89) at leaf scale, showing better correlations than using single-band reflectance (Fig. 3b). This highlighted the relevance of band combination to improve HM estimation. Another advantage of NDVI-like indices is their robustness to variations in measurement geometry, light conditions, and soil influence^30,64–67^. Thus, they are well-suited for application at larger scale of monitoring. In our case, the same optimized indices computed on field canopy measurements performed and on the airborne image still exhibited high correlations with HM contents in leaves (*r* ≤ − 0.76 for Cr, Cu and Ni, and *r* ≥ 0.87 for Zn) (Table 2). Some of the index values underwent slight variations among acquisition levels (i.e. leaf, canopy and image), but their overall relationship with HM remained almost intact on the calibration dataset (Fig. 5a–d).

**Table 2:** Summary of the performance of the optimized vegetation indices developed for predicting leaf heavy metal contents.

Heavy metal	Level	Calibration			Prediction		
		Optimized index	Equation	*r*	*r*	RMSE	RPD
Cr	Field leaf	(*ρ*_847_ − *ρ*_724_)/(*ρ*_847_ + *ρ*_724_)	*y* = − 11.48*x* + 3.83	− 0.82***	0.81***	0.27	2.02
	Field canopy		*y* = − 10.72*x* + 4.14	− 0.81***	0.78***	0.31	1.91
	image pixels		*y* = − 16.51*x* + 7	− 0.76***	0.75**	0.4	1.5
Cu	Field leaf	(*ρ*_719_ − *ρ*_532_)/(*ρ*_719_ + *ρ*_532_)	*y* = − 85.07*x* + 62.34	− 0.9***	0.89***	2.53	2.56
	Field canopy		*y* = − 96.94*x* + 69.12	− 0.89***	0.86***	3.22	2.06
	image pixels		*y* = − 191.54*x* + 129.93	− 0.85***	0.84***	3.05	2.17
Ni	Field leaf	(*ρ*_718_ − *ρ*_576_)/(*ρ*_718_ + *ρ*_576_)	*y* = − 6.57*x* + 5.59	− 0.84***	0.82***	0.28	1.98
	Field canopy		*y* = − 9.76*x* + 7.3	− 0.8**	0.78**	0.29	1.92
	image pixels		*y* = − 10.43*x* + 7.34	− 0.78**	0.77**	0.31	1.8
Zn	Field leaf	(*ρ*_646_ − *ρ*_553_)/(*ρ*_646_ + *ρ*_553_)	*y* = 315.29*x* + 245.4	0.89***	0.87***	12.88	2.14
	Field canopy		*y* = 271.29*x* + 194.1	0.9***	0.87***	11.53	2.46
	image pixels		*y* = 281.44*x* + 145.87	0.87***	0.85***	13.54	2.09

For each of the four metals, the results obtained at leaf, canopy and image levels are presented on the calibration and prediction sets.

*r* Pearson’s coefficient of correlation, *RMSE* root mean square error, *RPD* residual predictive deviation.

**p < 0.01; ***p < 0.001.

Once calibrated (step (2) of the methodology), the regression equations between each index and HM were applied for predicting metal contents, at all acquisition levels. This represented the step (3) of the methodology, the results of which are illustrated in Fig. 5e–h and detailed in Table 2. Each of the four HM were accurately retrieved at leaf, canopy and image pixel levels. This was highlighted by *r* values ≥ 0.75 and Residual Predictive Deviation (RPD) values close to 2. As expected, the predictions were more precise in the field (leaf and canopy levels) than on the image pixels. The best prediction results were obtained for Cu and Zn (*r* ≥ 0.84 and RPD ≥ 2.06), which were also present at higher concentration in leaves than Cr and Ni. Hence, these results demonstrate than HM uptake in leaves can be assessed accurately over industrial brownfields using field reflectance spectroscopy and airborne remote sensing.

### HM mapping over the brownfield

**Figure 6:**
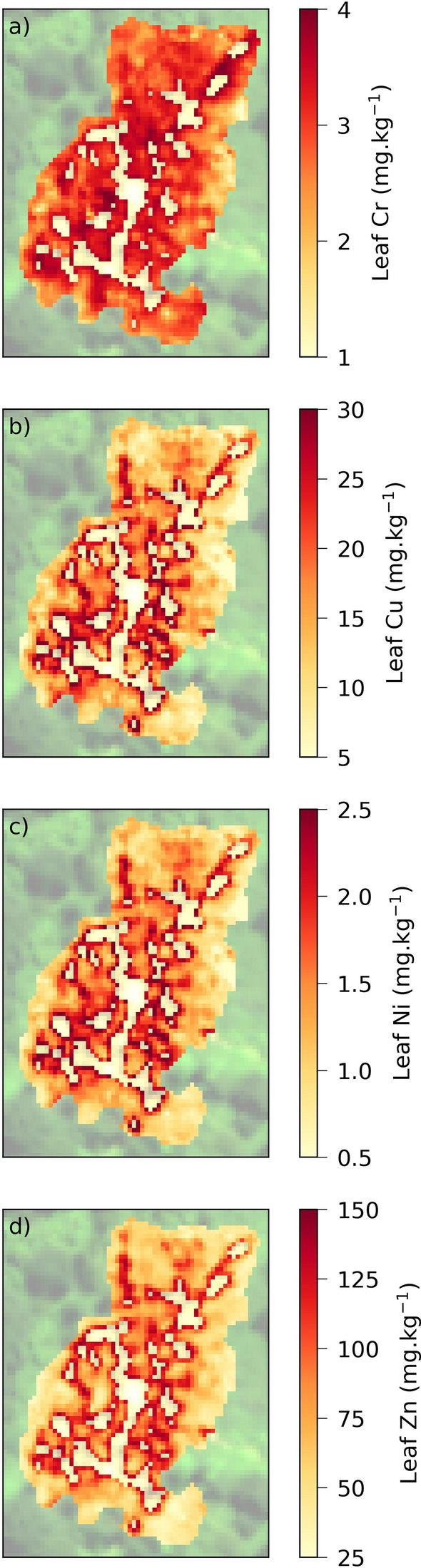
(**a**–**d**) Mapping of the four heavy metal contents in leaves over the brownfield site. These maps were obtained by computing the optimized vegetation indices and the regression equations listed in Table 2 on the airborne hyperspectral image, at 1 m spatial resolution.

The final step of the methodology consisted in mapping HM contents over the entire brownfield site. For this purpose, we applied the calibrated regression equations on the optimized vegetation index maps computed from the airborne image, at 1 m spatial resolution. The resulting mappings are presented in Fig. 6. The overall concentrations were in the range of the calibrated regression models, indicating that the leaves sampled in the field were representative of the site. The central part of the brownfield, which comprised the most polluted soils, also revealed higher metal uptake by bramble. Some of the high metal contents observed at the margin of bramble patches (leaf Cr close to 4 mg kg^−1^, and Cu and Zn contents > 28 and 143 mg kg^−1^, respectively) may be linked to mixed bare soil and vegetation in pixels. As the proportion of bare soil in pixels increases, the values of vegetation indices decrease. Since the indices were negatively correlated to HM concentrations in leaves, lower index values caused by bare soil led to overestimating metal concentrations at the margins of bramble patches. Differences in the spatial distribution of HM within the site were also noticed. The distribution of Cr in bramble leaves was the most homogeneous, followed by Ni, Zn and Cu, which was consistent with the variability of these metals in the soil (see the Supplementary Table S5).

### Future application in ecological risk assessment and phytoextraction monitoring

Based on previous work^32,33^, this study is the first to demonstrate that remote sensing can be used for mapping metal uptake in plants accurately, and thus for detecting hotspots of contamination with priority needs for remediation. These promising outcomes open perspectives of application in ecological risk assessment and phytoextraction monitoring. Field reflectance spectroscopy is recommenced for fast, non-destructive, and continuous monitoring of plant metal uptake. Canopy measurements offer a global view of the plant’s metal contents at different spatial scales (depending on the acquisition footprint), and do not require extensive data processing. Leaf measurements operate at very small scale (cm), providing a very precise assessment of metal uptake and translocation in leaves. It might be used also to determine the vertical distribution of HM in plants, which is a relevant information in phytoextraction. Another advantage of the leaf-clip device is to allow weather-independent measurements, so it can be used indifferently in greenhouse and field controlled trials and under natural conditions^30,58,68^.

The most innovative aspect of our methodology is probably its application to airborne images. In a perspective of operational application, very high spatial resolution is an essential criterion that must be satisfied to ensure accurate mapping of HM. There is a growing demand in developing cost-saving solutions based on Unmanned Aerial Vehicles (UAVs) imaging by field operators and environmental managers in industry. In this respect, we are convinced that our methodology could be easily adapted to UAV-embedded sensors. Along with previous work^32^, our study suggests that only few bands of the VNIR domain are needed to quantify leaf HM contents. These bands could serve for developing compact multispectral cameras devoted to monitor the uptake of metals by wild plant species and the effectiveness of phytoextraction trials from UAV. To achieve this, our methodology should be improved in order to fulfill the requirements of operational use. Since we focused on four common HM, one possible improvement would be to extend the mapping to other metals, especially As, Cd, Hg and Pb, which steadily persist in the soils of abandoned industrial facilities^1,30,69^. Some HM are generally correlated to each other in soils. So, further study should focus on unravelling the respective contribution of the different HM to leaf reflectance to better quantify these metals individually. Due to unfavorable growing conditions, brownfields usually exhibit low species richness^4,9^. In that sense, our study focused on a single species that predominates on urban and industrial sites under temperate regions. However, we encourage to extend our methodology to a wide range of plant species, especially hyperaccumulators^2,13^, to promote the use of field reflectance spectroscopy and remote sensing for monitoring phytoextraction trials, as a complement to existing techniques. This could be achieved by developing metal- and species-specific vegetation indices that can be applied to both field and image reflectance data.

A single-date mapping provides a snapshot of pollution uptake. However, leaf HM content is likely to vary in time for wild plant species, because of seasonal changes in environmental conditions and plant phenological stages. Likewise, it is expected to decrease over successive crop cycles in controlled phytoextraction trials. In both situations, a multi-temporal mapping becomes necessary. This represents a major challenge, as many varying environmental factors influence plant reflectance, independently from pollution. For example, seasonal changes in reflectance can prevail from those induced by HM, affecting the accuracy of estimates, according to recent field studies^9,32^. Thus, the performance of our methodology should be assessed on a multi-temporal basis, in a perspective of operational monitoring applications.

HM pollution is a current issue, and remote sensing offers a promising complement to existing techniques used in ecological risk assessment and phytoextraction monitoring. The development of compact and cost-saving cameras flourishes worldwide, and progressively draws the attention of field operators and environmental managers. Hence, although major improvements are yet required, remote sensing is intended to become an operational tool for monitoring soil pollution at large scale in the coming decades.

## Methods

### Study site

An old industrial brownfield located under temperate region was selected for this study (Fig. 1a,b). This site served as mud deposit for over 20 years to the oil and gas industry, accumulating hydrocarbon and HM pollution. The characteristics of the brownfield soil are presented in the Supplementary Table S1. In addition to pollution, its clayey soil imposes unfavorable growing conditions to plants due to the poor nutrient availability; most of the organic carbon coming from heavy C_10_–C_40_ hydrocarbon compounds, which are unavailable to plants. Only few species have established on the site, *Rubus fruticosus L*. (bramble), a shrubby and thorny plant being the predominant one. This species forms sparse but densely vegetated patches with limited development (Fig. 1c), and shows stress symptoms such as leaf discoloration and margin red pigmentation (Fig. 1d), which have been linked to soil pollution in previous studies^30,70^. *R. fruticosus* L. is a suitable model species for developing methodologies to monitor soil pollution from reflectance data^9,57^. It is also capable of accumulating HM in leaves^40–42^. Therefore, this species was selected for this study, which was carried out in July 2017.

### Soil and leaf heavy metal analysis

A mesh of 40 plots, uniformly distributed and colonized by bramble, was defined to cover most of the site. On each plot center, the geographical coordinates were acquired using a differential GPS. The upper soil layer (0–20 cm) was sampled and analyzed for HM as described in Qian et al.^4^, especially Cr, Cu, Ni and Zn (Table 1). This soil also contained high levels of petroleum hydrocarbons (see^9^). Young bramble leaves (*n* = 40) were also collected on the 40 brownfield plots. Cr, Cu, Ni and Zn contents from the leaves were analyzed by ICP-MS after acid digestion, as detailed in^71^. The results of these analyses are presented in Table 1. For each metal, the bioconcentration factor were computed according to the equation:

1 $$ {\text{BCF}} = \frac{{\left[ {\text{Heavy metal}} \right]_{{{\text{leaves}}}} }}{{\left[ {\text{Heavy metal}} \right]_{{{\text{soil}}}} }} $$

where [Heavy metal]_leaves_ and [Heavy metal]_soil_ stand for the HM concentration in bramble leaves and in the brownfield soil, respectively^4^. In addition, soil and leaf samples were collected and analyzed on an uncontaminated—control—site with similar soil characteristics (texture, pH, moisture, etc.) (see the Supplementary Table S2 for the detailed analysis). These samples served for calculating the Enrichment Factor (EF) of bramble leaves for the four metals, defined as:

2 $$ {\text{EF}} = \frac{{\left[ {\text{Heavy metal}} \right]_{{\text{brownfield leaves}}} }}{{\left[ {\text{Heavy metal}} \right]_{{\text{control leaves}}} }} $$

where [Heavy metal]_brownfield leaves_ and [Heavy metal]_control leaves_ are the HM contents in bramble leaves in the brownfield and the control site, respectively^40^.

### Field reflectance measurements

The spectral signatures of the leaves sampled on the brownfield were acquired in the [400:2500] nm domain using an ASD FieldSpec 4 Hi-Res spectroradiometer (Malvern Panalytical, Malvern, UK) attached with a leaf-clip. For this purpose, leaf radiance was measured on a black background panel using and internal light source, and converted to reflectance using a white reference calibration panel^58,72^. These data were used to develop the optimized vegetation indices intended to predict leaf HM contents. In addition, the spectral signature of bramble was also measured at the canopy level on the 40 sampled brownfield plots, using the same spectroradiometer and radiance-to-reflectance conversion procedure^57^. These measurements were performed between 11.30 am and 1.30 pm using a 25-mm wide fore optics fixed 45 cm above the canopy at nadir, resulting in a 20-cm acquisition footprint^30^.

### Image acquisition and preprocessing

On July 5, 2017, an airborne hyperspectral image was acquired over the brownfield at 1.15 pm, 2103 m above sea level under cloudless conditions (Fig. 1a,b), using a VNIR-1600 HySpex sensor (Norsk Elektro Optikk AS, Lørenskog, Norway) with 1-m spatial resolution and 5.2 nm spectral resolution in the [414:992] nm VNIR domain. The image was radiometrically corrected using standard materials with known reflectance. Then, the radiance image was atmospherically corrected using the Empirical Line Method^57,73,74^ to obtain surface reflectance, owing to the lack of knowledge about the local industrial atmosphere composition. The pixels corresponding to the field plots were then extracted from the image. Finally, a Savitzky-Golay smoothing was applied on all the reflectance data (field and image) to enhance the signal-to-noise ratio^75^.

### Leaf HM mapping

To achieve leaf HM mapping, the proposed methodology relied on four successive steps, namely (1) Development of optimized vegetation indices, (2) Calibration of index-HM regression equation, (3) Prediction and assessment, and (4) Mapping of leaf HM. The first step consisted in developing optimized vegetation indices for each of the four metals (Cr, Cu, Ni and Zn). For this purpose, we selected two thirds of the leaf reflectance and HM data (*n* = 26) using the Kennard-Stone algorithm^9,76^, and we focused on the VNIR domain (400–1000), which was common to all the datasets. First, the relationship between each single band (i.e. wavelength) and the four metal contents was evaluated through the Pearson correlation of coefficient (*r*), after verifying that the data followed normal distribution (Shapiro–Wilk test, *p* > 0.05). This helped identifying the most relevant spectral regions for each metal. Then, we tested simple and normalized vegetation indices (see^26,77^ for the index formulas). Here, we describe only the results for the Normalized Difference Vegetation Indices (NDVI-like indices), which describe the difference of reflectance between two wavelengths. These indices provided the best results regardless of the metal and the study level (field or image). Based on the traditional NDVI^78^, the general equation for these indices is:

3 $$ {\text{NDVI}} = \frac{{\rho_{i} - \rho_{j} }}{{\rho_{i} + \rho_{j} }} $$

where *ρ*_i_ and *ρ*_j_ are the reflectance values at wavelength i and j, respectively. This index—which ranges from − 1 to 1—originally exploits reflectance in the red and near-infrared wavelengths^79,80^. It shifts toward lower values as vegetation becomes sparse or stressed and the soil influence increases. Here, we varied the red/near-infrared wavelengths to find the best combination. Based on Eq. (3), all the possible VNIR band combinations (*ρ*_i_, *ρ*_i_) were computed and their correlation with the four metal contents was assessed at the significance level of *p* < 0.05. Several regression equations were tested (e.g*.* linear, exponential, logarithmic) and, except for soil contamination^9,26^, linear relationship provided the highest correlations. Finally, the best band combination (highest ***|r|***, *p* < 0.05) was selected for each metal, and used in the following steps.

At the end of the first step, the coefficients of linear regression equations between indices and HM were obtained on the leaf dataset. So, in the second step, they were adjusted independently at canopy and image levels, using again two thirds of the data. Then, these equations were used to predict leaf HM contents on the remaining third of the dataset (*n* = 14), at leaf, canopy and image levels. This represented the third step of the methodology. The quality of predictions was assessed using the Pearson’s *r*, the Root Mean Square Error (RMSE):

4 $$ {\text{RMSE}} = \sqrt {\frac{{\mathop \sum \nolimits_{j = 0}^{n} \left( {y^{\prime}_{j} - y_{j} } \right)^{2} }}{n}} $$

where *y*_j_*′* and *y*_j_ denote the predicted and measured metal contents in leaves, and the Residual Predictive Deviation (RPD), defined as follows:

5 $$ {\text{RPD}} = \frac{\sigma }{{{\text{RMSE}}}} $$

where σ is the standard-deviation of leaf metal concentration in the dataset. The higher the RPD, the better the model performance. It is commonly accepted that RPD values above 2 indicate good predictions, especially in the context of pollution monitoring^27,57^. Finally, the optimized indices were used to map the metal content of bramble leaves over the entire brownfield from the airborne image. To limit the mapping to the extent of the site, we created a shape of the brownfield from GPS measurements performed in the field. We also isolated the vegetation by applying a red-near-infrared NDVI threshold of 0.3. This procedure was applied to the four heavy metals. All the data processing was performed using Python 3.7.6 (https://www.python.org/) and Statsmodels^81^, Scipy^82^, Scikit-Learn^83^, and Rasterio packages.

## Supplementary Information

Supplementary Information

## Data Availability

The datasets generated and analyzed during the current study are available from the corresponding author on reasonable request. Only the georeferenced airborne image is not available on request because of the confidentiality of the site’s location.
