# Supplementary material for: Mapping leaf metal content over industrial brownfields using airborne hyperspectral imaging and optimized vegetation indices
Source: Sci Rep. 2021 Jan 7;11:2. doi: 10.1038/s41598-020-79439-z (PMC7791056; doi:10.1038/s41598-020-79439-z)
Supplement: Supplementary file 1 — Supplementary Information [file 41598_2020_79439_MOESM1_ESM.pdf]

Supplementary Information

Mapping leaf metal content over industrial brownfields using airborne hyperspectral imaging and optimized vegetation indices

Guillaume Lassalle[a, b, c], *Sophie Fabre*[a], *Anthony Credoz*[b], *Rémy Hédacq*[b], *Dominique Dubucq*[d], *Arnaud Elger*[c,*]

[a] Office National d'Études et de Recherches Aérospatiales (ONERA), Toulouse, France

[b] TOTAL S.A., Pôle d'Études et de Recherches de Lacq, Lacq, France

[c] Laboratoire Écologie Fonctionnelle et Environnement (EcoLab), Université de Toulouse, CNRS, INPT, UPS, Toulouse, France

[d] TOTAL S.A., Centre Scientifique et Technique Jean-Féger, Pau, France

Table S1. Characteristics of the soil sampled on the brownfield site.

Parameter	Mean value
pH	6.4
Cation Exchange Capacity ($cmol.kg^{-1}$)	9.78
Moisture (%)	21.80
TOC ($g.kg^{-1}$)	139
TN ($g.kg^{-1}$)	2.44
Exchangeable K ($mg.kg^{-1}$)	0.0595
Exchangeable P ($mg.kg^{-1}$)	0.005

Table S2. Heavy metal contents observed in the soil and in the leaves of *R. fruticosus* L on the control site. For each heavy metal, the geochemical background is also specified.

	Range	Mean (± SD)	Geochemical background
Soil ($mg.kg^{-1}$):			
Cr	16 - 22	19 ± 4.24	39
Cu	15 - 27	21 ± 8.49	41
Ni	11 - 16	13.5 ± 3.54	40
Zn	54 - 96	75 ± 29.7	704
Leaves ($mg.kg^{-1}$):			
Cr	0.43 - 0.83	0.63 ± 0.13	
Cu	7.7 - 8.11	7.91 ± 0.14	
Ni	1.31 - 1.73	1.52 ± 0.14	
Zn	41.17 - 42.96	41.96 ± 0.67	

Table S3. Review of heavy metal uptake and accumulation in *Rubus fruticosus* L. growing on polluted soils. (BCF: Bioconcentration Factor; TF: Translocation Factor; EF: Enrichment Factor.)

Metal	Range in soil ($mg.kg^{-1}$)	Range in leaves ($mg.kg^{-1}$)	Range BCF	Range TF	Range EF	Source
Cu	12.89 - 12.89	12.46 - 12.46	0.967 - 0.967	0.663 - 0.663	-	[1]
	6.02 - 348.12	1.9 - 5.1	0.015 - 0.015	-	-	[2]
	9.7 - 2210	20 - 510	0.064 - 2.062	0.526 - 1.538	3.2 - 27.7	[3]
	26 - 990	13 - 265	0.046 - 10.192	0.2 - 5.638	-	[4]
Ni	12.43 - 12.43	5.1 - 5.1	0.015 - 0.015	-	-	[1]
	14.2 - 53.4	2.2 - 8	0.065 - 0.563	0.236 - 1.429	0.5 - 1.7	[3]
Zn	36.52 - 36.52	20.24 - 20.24	0.554 - 0.554	0.398 - 0.398	-	[1]
	4.87 - 482.79	29.1 - 75	-	-	-	[2]
	45.3 - 307	20 - 195	0.137 - 0.83	0.333 - 1.471	1.6 - 9.3	[3]
	532 - 2200	93 - 400	0.169 - 0.318	0.538 - 1.176	-	[4]

Table S4. Environmental recommendations of the European Union (EU) and the French National Institute for Industrial Environment and Risks (INERIS) for heavy metal concentrations in soils. The concentrations are given in $mg.kg^{-1}$. (PNEC: Predicted No Effect Concentration.)

	EU standard	INERIS (PNEC)
Cr	150	Cr(VI): 0.035 – Cr (III): 3.2
Cu	140	2.7
Ni	75	4.3
Zn	300	26 (60 for plants)

Table S5. Statistics of heavy metals contents in the leaves of *R. fruticosus* L. obtained from the airborne hyperspectral image over the whole brownfield site.

	Min	Max	Mean	Std	CV (%)
Heavy metal ($mg.kg^{-1}$):					
Cr	1.08	3.93	2.94	0.53	18.03
Cu	5.50	28.84	16.61	7.10	42.75
Ni	0.56	2.48	1.44	0.54	37.50
Zn	25.57	143.80	77.45	29.93	38.64

References:

1. Alagić, S., Tošić, S. B., Dimitrijević, M. D., Petrović, J. V. & Medić, D. V. The Characterization of Heavy Metals in the Grapevine (Vitis vinifera) Cultivar Rkatsiteli and Wild Blackberry (Rubus fruticosus) from East Serbia by ICP-OES and BAFs. *Commun. Soil Sci. Plant Anal.* **47**, 2034–2045 (2016).

2. Dorrington, V. H. & Pyatt, F. B. Some aspects of tissue accumulation and tolerance to available heavy metal ions by Rubus Fruticosus L., A Colonizer of spoil tips in S.W. England. *Int. J. Environ. Stud.* **20**, 229–237 (1983).

3. Nujkić, M. M., Dimitrijević, M. M., Alagić, S. Č., Tošić, S. B. & Petrović, J. V. Impact of metallurgical activities on the content of trace elements in the spatial soil and plant parts of Rubus fruticosus L. *Environ. Sci. Process. Impacts* **18**, 350–360 (2016).

4. Yoon, J., Cao, X., Zhou, Q. & Ma, L. Q. Accumulation of Pb, Cu, and Zn in native plants growing on a contaminated Florida site. *Sci. Total Environ.* **368**, 456–464 (2006).
